# Prevalence and Clinicopathological Characteristics of *HER2* and *BRAF* Mutation in Chinese Patients with Lung Adenocarcinoma

**DOI:** 10.1371/journal.pone.0130447

**Published:** 2015-06-23

**Authors:** Ling Shan, Tian Qiu, Yun Ling, Lei Guo, Bo Zheng, Bingning Wang, Wenbin Li, Lin Li, Jianming Ying

**Affiliations:** Department of Pathology, Cancer Hospital, Chinese Academy of Medical Sciences, Peking Union Medical College, Beijing, China; University of Central Florida, UNITED STATES

## Abstract

**Aims:**

To determine the prevalence and clinicopathological characteristics of *BRAF* V600E mutation and *HER2* exon 20 insertions in Chinese lung adenocarcinoma (ADC) patients.

**Methods:**

Given the fact that the driver mutations are mutually exclusive in lung ADCs, 204 *EGFR*/*KRAS* wild-type cases were enrolled in this study. Direct Sanger sequencing was performed to examine *BRAF* V600E and *HER2* exon 20 mutations. The association of *BRAF* and *HER2* mutations with clinicopathological characteristics was statistically analyzed.

**Results:**

Among the 204 lung ADCs tested, 11 cases (5.4%) carried *HER2* exon 20 insertions and 4 cases (2.0%) had *BRAF* V600E mutation. *HER2* mutation status was identified to be associated with a non-smoking history (p<0.05). *HER2* mutation occurs in 9.4% of never smokers (10/106), 8.7% of female (8/92) and 2.7% of male (3/112) in this selected cohort. All four *BRAF* mutated patients were women and three of them were never-smokers. No *HER2* mutant patients harbor *BRAF* mutation.

**Conclusions:**

*HER2* and *BRAF* mutations identify a distinct subset of lung ADCs. Given the high prevalence of lung cancer and the availability of targeted therapy, Chinese lung ADC patients without *EGFR* and *KRAS* mutations are recommended for *HER2* and *BRAF* mutations detection, especially for those never smokers.

## Introduction

Lung cancer is the leading cause of cancer-related death worldwide [1]. Adenocarcinoma (ADC), the most common type of lung cancer, is diagnosed in 1 million patients each year [2]. Targeted therapies have been succeeded in a subset of lung ADC patients with driver oncogenic mutations [3,4]. Currently a higher than 50% estimated frequency of actionable oncogenic drivers have been identified in lung ADCs. Sensitizing *EGFR* mutations occur in 30% -50% of Asian lung ADC patients, who are potential responders for EGFR tyrosine kinase inhibitors (TKIs) treatment. *ALK* and *ROS1* rearrangements, targeted by crizontinib, appear in approximately 6%-10% of lung ADC patients. Testing for somatic *EGFR* mutations and *ALK* rearrangements is now in clinical routine for advanced lung ADC patients. Another two actionable targets, *BRAF* and *HER2* mutations, have been identified in approximately 3% and 2% of lung ADC patients, respectively [5–7]. Vemurafenib, the selective BRAF kinase inhibitors, has been approved and succeed for the treatment of melanoma patients harboring *BRAF* V600E mutation. It provided a rationale for testing *BRAF* mutation in lung ADC patients. Very recently, dramatic response of Vemurafenib and Dabrafenib treatment has been observed in lung ADC patients with *BRAF* V600E mutation [8–10]. Meanwhile, *HER2* exon 20 insertions in lung ADC patients were identified to indicate efficacy of HER2-targeted drugs, i.e. trastuzumab and afatinib [11,12]. As a result, the importance of screening for *BRAF* and *HER2* mutations in lung ADC patients is recognized in clinical practice. However, as only a few patients would harbor the *BRAF* and *HER2* mutations, it is not plausible to examine these mutations in all lung ADC patients. Although efforts have been made to identify the clinicopathological factors of the lung ADC patients harboring the *BRAF* or *HER2* mutations, the studies were performed predominately on white and Japanese patients [7,11,13–16]. For Chinese lung ADC patients, *HER2* and *BRAF* mutations have been selectively examined in never-smokers [17–19]. Given the fact that the epidemiology and clinical behaviors of lung cancer is different between East Asians and Caucasians [20], we examined the *BRAF* V600E mutation and *HER2* exon 20 insertions in Chinese lung ADC patients in order to determine the frequency of these two mutations and identify their clinicopathological characteristics.

## Materials and Methods

### Patient selection

All included patients had received curative surgery and diagnosed as primary lung ADC. Mutation testing of *EGFR* and *KRAS* genes had been routinely performed for all the samples at the Cancer Hospital, Chinese Academy of Medical Sciences (CAMS), Beijing, China. Hematoxylin and eosin-stained (HE) sections of formalin-fixed paraffin-embedded (FFPE) tissue were reviewed for each sample to identify the section with the highest tumor density (at least 50% tumor content). Genomic DNA was extracted using the QIAamp DNA Mini Tissue kit (Qiagen, Germany) following the manufacturer’s standard protocol. Clinical testing for *EGFR* was carried out using quantitative real-time PCR (qRT-PCR) (Beijing ACCB Biotech Ltd., China) for the detection of small indels in exons 19 and 20, the G719X mutation in exon18, the T790M mutation in exon 20 and the L858R and L861Q mutation in exon 21. *KRAS* testing was performed using qRT-PCR for the detection of the G12X and G13D mutations (Beijing ACCB Biotech Ltd., China). All DNA samples were kept in -80°C freezer after the mutation testing for long-term storage and the *EGFR* and *KRAS* mutation status were recorded electronically. According to the record, 215 cases were negative for *EGFR* (exons 18–21) and *KRAS* (G12 and G13) mutations between January 1, 2008 and December 31, 2012. Two hundred and four cases had enough stored DNA for *HER2* and *BRAF* mutation analysis. The clinicopathological records of these patients were retrospectively collected from the Department of Pathology, CAMS, including sex, age, smoking history, tumor size, histological subtype, pT, pN and pTNM stages. Two of the most predominant histological subtypes for each tumor were used to further analysis. This study is retrospective and the data were analyzed anonymously. No images and private information of the patients were released. The Institute Review Board of the Cancer Hospital, CAMS, agreed to waive the need for consent for this study and approved the study protocol.

### *BRAF* and *HER2* mutation analysis

*BRAF* V600E and *HER2* exon 20 mutation analysis was carried out using direct Sanger sequencing. Briefly, the *BRAF* V600E mutation was examined though amplifying the exon 15 using forward primer, 5’- TCATAATGCTTGCTCTGATAGGA-3’ and reverse primer, 5’- GGCCAAAAATTTAATCAGTGGA-3’. The entire coding region of *HER2* exon 20 was amplified using forward primer, 5’- GCCATGGCTGTGGTTTGTGATGG-3’ and reverse primer, 5’-ATCCTAGCCCCTTGTGGACATAGG-3’. The Refseq accession number for *HER2* gene analyzed in this study is NM_001289937.

### Statistical analysis

The statistical analysis of the tumors’ size and age was carried out using Student’s t tests. The values are shown as mean ± SD. The relationship between *HER2* mutation and clinicopathological variables was analyzed with the chi-square test. Statistical significance was defined as *p* < 0.05.

## Results

### *BRAF* and *HER2* mutations

**Fig 1:**
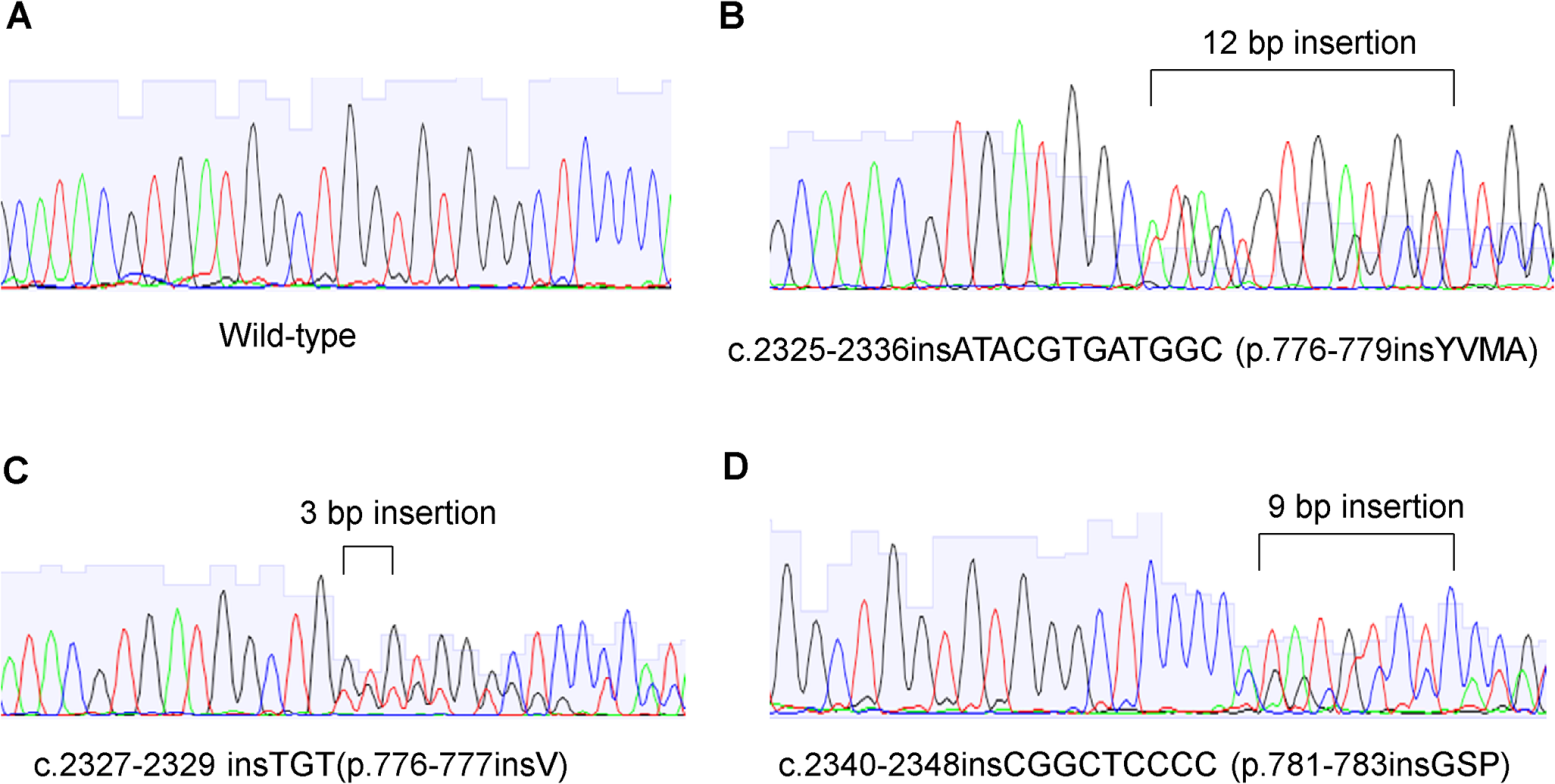
Sanger sequencing reads demonstrating mutational patterns of *HER2* exon 20. (A) Wild-type sequence. (B) A 12-bp duplication/insertion of the amino acid sequence YVMA between codon 776 and 779. (C) A 3-bp insertion of the amino acid sequence VC at codon 776. (D) A 9-bp insertion of the amino acid GSP between codon 781 and 783.

According to the known mutually exclusive nature of driver mutations, 204 lung ADC cases without activating *EGFR* (exons 18–21) and *KRAS* (G12 and G13) mutations were selected for *BRAF* and *HER2* mutation analysis. Among the 204 lung ADCs tested, 11 cases (5.4%) were with *HER2* exon 20 insertions and 4 cases (2.0%) were identified with *BRAF* V600E mutation. All *HER2* mutations in exon 20 were in-frame insertions ranged from 3 to 12 bp between codon 775 and 780 (**Fig 1**). The 12 bp insertion was the most common mutation (45.5%, 5/11). All these cases showed a duplication/insertion of 4 amino acids (YVMA) at codon 775. The 3 bp insertion at codon 776 was the second most common mutation (36.4%, 4/11). This insertion resulted in a replacement of codon 776 (G) by 2 amino acids (VC). Two cases (18.2%, 2/11) were identified with 9 bp insertion and resulted in a duplication/insertion of 3 amino acids (GSP) at codon 780.

### Clinicopathological characteristics of patients with *HER2* or *BRAF* mutations in *EGFR/KRAS* wild-type lung ADCs

The clinicopathological characteristics of the *HER2* and *BRAF* mutations in *EGFR*/*KRAS* wild-type lung ADCs are summarized in **Table 1**. Compared to *HER2* wild-type group, although not significant, the patients with *HER2* mutations tend to be more in women (8/11) than men (3/11). All *BRAF* mutations occurred in women (4/4) in this cohort. In 92 female patients, 13% of them carried either *HER2* or *BRAF* mutations. Patients with *HER2* mutations were more likely to be never smoker (90.9%, 10/11) compared to ever smoker (9.1%, 1/11) (p<0.05). In 106 never smokers, 12.3% of patients carried either *HER2* or *BRAF* mutant tumors. There were no significant differences of *HER2* or *BRAF* mutations regarding tumor size, pT, pN factors or pTNM stages. The predominant histological subtype of *HER2* mutated patients was acinar in 10/11 (90.9%) of cases, solid pattern in 6/11 (54.5%), papillary in 4/11 (36%), and micropapillary in 4/11 (36%). No *HER2* mutant patients harbor *BRAF* mutation.

**Table 1:** Clinicopathologic comparisons between *HER2* and *BRAF* mutation positive and negative Lung adenocarcinomas.

	Overall	HER2 mutation	HER2 wild-type		BRAF mutation	BRAF wild-type	
	n = 204	n = 11 (5.4%)	n = 193 (94.6%)	*P* value	n = 4 (2.0%)	n = 200 (98.0%)	*P* value
**Age**	Mean±SD	55.4±5.9	58.4±10.2	0.861	66.8±6.3	58.0±10.1	0.735
	Median	56	58		67	58	
	Range	44–62	25–81		59–74	25–81	
**Sex**	Male	3	109	0.114	0	112	0.085
	Female	8	84		4	88	
	Nonavailable						
**Smoking**	Never smoker	10	96	0.035	3	103	0.785
	Ever smoker	1	83		1	83	
	Nonavailable	0	14		0	14	
**Tumour size (mm)**	< = 2	2	48	1.000	0	50	0.424
	2–5	8	112		3	117	
	>5	1	32		1	32	
	Nonavailable	0	1		0	1	
**pT status**	pT1	1	20	1.000	0	21	1.000
	pT2	6	105		4	107	
	pT3-T4	2	25		0	27	
	Nonavailable	2	43		0	45	
**pN status**	pN0	4	78	0.294	2	80	1.000
	pN1	1	43		0	44	
	pN2-3	6	58		2	62	
	Nonavailable	0	14		0	14	
**Clinical stage**	I	2	69	0.074	2	69	1.000
	II	1	47		0	48	
	III-IV	8	74		2	80	
** **	Nonavailable		3		0	3	

## Discussion

*HER2* and *BRAF* genes represent relatively new biomarkers for NSCLC. *HER2* (also known as *EGFR2*, *ERBB2* or *NEU*) belongs to the ERBB family. Like other family members, HER2 is structurally constituted by three domains: an extracellular domain responsible for ligand binding and homo/heterodimers formation, a transmembrane domain that makes a single pass through the plasma membrane and a tyrosine kinase (TK) domain responsible for activation of two key signaling pathways, namely, the RAS/RAF/MAPK pathway, which stimulates proliferation, and the PI3K/Akt pathway, which promotes tumor cell survival. *HER2* mutations occur in the TK domain to cause a conformational change, which lead to an increased kinase activity compared to the wild-type form. Both *in-vitro* and *in-vivo* studies have confirmed the oncogenic potential of these mutations [21–23]. Given the fact that the driver mutations are mutually exclusive in lung ADCs [13,24], we selected 204 cases negative for the activating *EGFR* and *KRAS* mutations in this study. The frequency of *HER2* exon 20 mutations was 5.4% in this cohort. The incidence of *HER2* mutations has been reported previously to range from 1% to 6% in NSCLC, and the vast majority of *HER2* mutations were represented by a 12 bp duplication/insertion of the amino acid sequence YVMA in exon 20 at codon 776 [7,11,13,25,26]. The highest frequency described was 5% in *EGFR*/*KRAS* wild-type and 6% in *EGFR*/*KRAS*/*ALK* wild-type populations, respectively [13]. In Chinese lung ADC patients, *HER2* mutation was identified in 6% of never-smokers [18]. Although the incidence of *HER2* mutation in *EGFR*/*KRAS* wild-type NSCLC patients in this study was similar to others in white patients, there is a difference regarding the frequencies of different mutation subtypes. The most common mutation subtype of *HER2* was still the 12 bp duplication/insertion of the amino acid sequence YVMA in exon 20 at codon 776 in this study, however, the frequency of this mutation subtype (45.5%) was lower compared to other studies in white patients (~80%). The result in this study could not compare to those performed on Asian lung ADC patients, in which the detailed information of insertion site in exon 20 of *HER2* gene was absent [7,17,18,27]. *In vitro* studies have shown that tumor cells harboring the most prevalent *HER2* ^YVMA^ are able to activate EGFR in a ligand-independent fashion and irrespective of the presence of an activating *EGFR* mutation [22,28]. In addition, the tumor cells harboring *HER2* ^YVMA^ mutations have been demonstrated to be resistant to reversible EGFR-TKIs such as gefitinib and erlotinib, while they remain sensitive to HER2 and dual EGFR/HER2 inhibitors [28]. In the largest published series, Mazieres *et al*. reported an impressive response rate of nearly 60% for HER2^YVMA^ mutation positive subjects receiving trastuzumab and chemotherapy [11]. However, it is still not clear if other two mutations, *HER2* ^V^ and *HER2* ^GSP^, would benefit from trastuzumab. Thus, further clinical trials are required.

Based on published studies, the presence of *HER2* mutations seems associated with female gender and never smokers in lung ADC patients [11,29,30]. In this study, *HER2* mutations were confirmed to be associated with never smokers (90.9%, 10/11) (p<0.05) and more in women than men (72.7% vs. 27.3%). In this *EGFR*/*KRAS* wild-type lung ADC patient cohort, *HER2* mutation occurs in 9.4% of never smokers (10/106), 8.7% of female (8/92) and 2.7% of male (3/112).

*BRAF* mutations have been reported in 2% to 4.9% of white patients and less than 1% of Asian patients with NSCLC [6,14,19,25,31,32]. In this selected patient cohort, the frequency of *BRAF* mutation only reached to 2% (4/204). Therefore, it seems that *BRAF* mutation is more common in the white NSCLC patients than in the Asian. Due to the low frequency, there is no agreement so far regarding *BRAF* mutations associated clinicopathological characteristics including sex and smoking history. In a study with the largest series of patients with *BRAF* mutant lung cancers, most patients were identified to be heavy smokers [33]. However, in this study, all four *BRAF* mutated patients are female and three of them are never-smokers.

In summary, *HER2* and *BRAF* mutations identify a distinct subset of lung ADCs. In Chinese *EGFR*/*KRAS* wild-type lung ADCs, 7.4% of the patients and 12.3% of never smokers carry *HER2* or *BRAF* mutations. Given the high prevalence of lung cancer and the availability of targeted therapy, Chinese lung ADC patients without *EGFR* and *KRAS* mutations are recommended for *HER2* and *BRAF* mutations detection, especially for those never smokers.
